# Plant-Derived Exosome-like Nanoparticles: A Comprehensive Overview of Their Composition, Biogenesis, Isolation, and Biological Applications

**DOI:** 10.3390/ijms252212092

**Published:** 2024-11-11

**Authors:** Ajia Sha, Yingyong Luo, Wenqi Xiao, Jing He, Xiaodie Chen, Zhuang Xiong, Lianxin Peng, Liang Zou, Bingliang Liu, Qiang Li

**Affiliations:** Key Laboratory of Coarse Cereal Processing, Ministry of Agriculture and Rural Affairs, Sichuan Engineering & Technology Research Center of Coarse Cereal Industrialization, School of Food and Biological Engineering, Chengdu University, No. 2025, Chengluo Avenue, Longquanyi District, Chengdu 610106, China; shaajia19980108@126.com (A.S.); lyy1478963@126.com (Y.L.); xwq990713@126.com (W.X.); hejing1244177474@126.com (J.H.); cxd0512@126.com (X.C.); xiongzhuang2000@126.com (Z.X.); penglianxin@cdu.edu.cn (L.P.); zoulian@cdu.edu.cn (L.Z.)

**Keywords:** PELNs, plants, nanoparticles, isolation and purification, functional and application

## Abstract

Plant-derived exosome-like nanoparticles (PELNs) are a type of membranous vesicle isolated from plant tissues. They contain proteins, lipids, nucleic acids, and other components. PELNs are involved in the defensive response to pathogen attacks by exerting anti-inflammatory, antiviral, antifibrotic, and antitumor effects through the substances they contain. Most PELNs are edible and can be used as carriers for delivering specific drugs without toxicity and side effects, making them a hot topic of research. Sources of PELNs are abundantly, and they can be produced in high yields, with a low risk of developing immunogenicity in vivo. This paper summarizes the formation, isolation, and purification methods; physical properties; and composition of PELNs through a comprehensive literature search. It also analyzes the biomedical applications of PELNs, as well as future research directions. This paper provides new ideas and methods for future research on PELNs.

## 1. Introduction

All cells, both prokaryotic and eukaryotic, can release extracellular vesicles (EVs) [1,2,3]. EVs can be broadly classified into two categories: ectosomes and exosomes. Exosomes are EVs with diameters ranging from approximately 30 to 150 nm [4]. In 1983, scientists noticed microvesicles being released from reticulocytes into the external environment. These were then termed “exosome-like nanoparticles (ELNs)”, representing a type of secretion from cells [5,6]. With the discovery of ELNs in animal cells, a growing body of research has found that vesicles similar to ELNs are also produced in plants. Plant-derived exosome-like nanovesicles are commonly referred to as PELNs. In the 1960s, studies showed that carrot cells could secrete vesicles [7]. In 2009, Regente et al. found that carrot cells could secrete vesicles [8]. Scientists isolated the extracellular fluid of sunflower seeds, and through transmission electron microscopy and proteomics analysis, they verified the presence of PELNs, thus initiating the exploration of PELNs. In the years that followed, a multitude of studies were conducted to isolate PELNs from various edible fruits and Chinese medicinal materials, such as ginger [9], grapes [10], and ginseng [11].

Some studies have suggested that PELNs are similar to ELNs secreted by animal cells, containing proteins, nucleic acids, lipids, and small active molecules. Mammalian-cell-derived ELNs have been utilized in many biomedical applications in recent years, including drug delivery, disease diagnosis, and tissue reconstruction. Few studies have looked into the source, makeup, and role of PELNs. Still, studies have demonstrated that they can act as an intercellular communication device between plants and regulate their innate immunity. For example, those containing resveratrol and solid lipid nanoparticles from grape extract can be used to treat Alzheimer’s disease (AD) [12]. Grapefruit-derived nanovesicles (GDNs) were found to be selectively taken up by intestinal macrophages and had a beneficial effect on mice with dextran sodium sulfate (DSS)-induced colitis, indicating that particular PELNs could act as mediators in the intestinal tract. These PELNs can act as immunomodulatory agents, regulating intestinal macrophage homeostasis. PELNs could potentially be used to deliver small molecules orally, reducing inflammation in humans affected by various illnesses [13]. Natural nanocarriers (TFENs) from *Camellia sinensis* specifically trigger breast tumor apoptosis and inhibit lung metastasis [14]. Bitter-melon-derived ELNs can protect myocardial cells from radiation damage by decreasing DNA damage and restoring mitochondrial dysfunction [15].

PELNs from different plant sources, such as licorice, cinnamon, kudzu, ginger root, peony, and astragalus, have been shown to have immunostimulatory effects when used in herbal medicine. These plant sources include Chinese herbal medicines such as licorice, cinnamon bark, *Pueraria lobata* root, ginger root, peony, and astragalus [16]. Edible PELNs do not have any toxicity. There are many medicinal plants found in nature. PELNs can be isolated from the fruits, leaves, seeds, and roots of the whole plant, as well as from differentiated tissues such as the media, stem cells, waste materials, shells, or phloem of the whole plant [17]. PELNs have the potential for large-scale production, and due to their intrinsic characteristics and ability to carry other compounds, such as drugs or small RNA (sRNA) molecules, they offer great potential for exploring the mechanisms of action in disease treatment, making them invaluable for both basic and clinical research [18].

This paper outlines the content, isolation techniques, characterization methods, preservation techniques, and functions of PELNs, with a focus on the potential applications of PELNs in clinical diseases, including as antiviral, antitumor, and antifibrotic agents, drug delivery, and in the promotion of antimelanin effects and cell proliferation. This paper also analyzes and predicts the future research directions of PELNs, providing a reference for a thorough analysis of the mechanism of PELNs.

## 2. Biogenesis of PELNs

**Figure 1:**
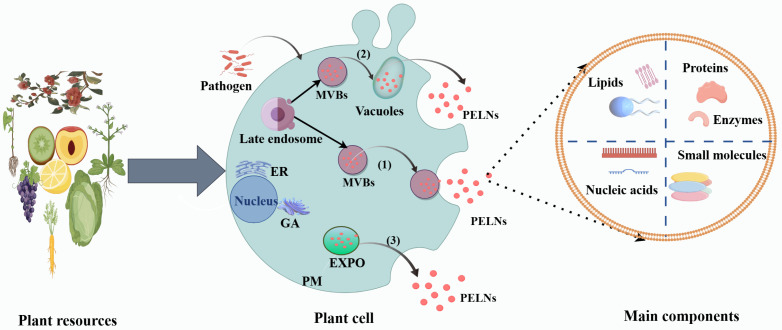
Sources, biogenesis, and contents of PELNs. Route (1) shows the vacuole fusion with the PM to release the remaining ILVs, which are obtained from MVBs. Route (2) depicts the MVB fusion with the PM to release the ILVs as PELNs. Route (3) shows the EXPO secretion. Abbreviations: PM, cytoplasmic membrane; ER, endoplasmic reticulum; GA, Golgi apparatus; MVBs, multivesicular bodies; EXPO, extracellular-positive organelle. Partly based on literature mapping by Cui et al. [24] and Cong et al. [20].

Compared with animal ELNs, there is a lack of research on PELNs, especially in terms of understanding their mechanisms of occurrence. It is now generally accepted that there are three possible pathways for the formation of PELNs: the multivesicular body (MVB) pathway, the EXPO (exocyst-positive organelle) pathway, and the vacuole pathway (Figure 1). MVBs are a class of late endosomes formed by endocytosis, which occurs after the cytoplasmic membrane concaves inwards [19]. The lumen of MVBs contains multiple tiny vesicles (intraluminal vesicles; ILVs). MVBs fuse with the cytoplasmic membrane and release their contents (ILVs) into the extracellular environment; the released vesicles are termed PELNs. The MVB pathway is considered to be the main pathway for the occurrence of PELNs. Another source of PELN occurrence is the EXPO pathway [20]. Wang et al. identified a special organelle in *Arabidopsis thaliana* (*A. thaliana*) cells that presents a spherical double-membrane structure similar to that found in autophagosomes. This organelle can fuse with the plasma membrane to release single-membrane vesicles [21]. It is now widely accepted by researchers that the EXPO pathway may be mediated through the fusion of EXPO with the plasma membrane after its formation in the cell, followed by the release of PELNs into the extracellular environment [22]. In recent years, more and more scholars have found that, in addition to the MVB and EXPO pathways, vesicles in plant cells may also be involved in the formation of PELNs. Cui et al. found that the central vesicle in plant cells originates from the MVB to SV transition and subsequent SV fusion [22]. In addition, Hatsugai et al. found that when *A. thaliana* is attacked by pathogenic bacteria, the *A. thaliana* vesicle membrane can fuse with the PM, resulting in the release of the vesicle contents (including various proteases) into the extracellular space [23]. It is now believed that in the vesicular pathway, MVBs may first release their contents (ILVs) into the vesicles, and then further release PELNs into the extracellular environment via fusion of the vesicles with the PM [24]. Since the fusion of vesicles with the PM to release PELNs has not yet been directly observed, the ILVs in the vesicles may be degraded before being released into the extracellular space. This speculation needs to be further verified.

## 3. Contents of PELNs

PELNs contain lipids, microRNAs (miRNAs), proteins, small molecules, and other bioactive components [25,26,27,28]. Table 1 summarizes the composition of and analysis techniques for PELNs from a number of plants.

Lipids are the main components of PELNs and play an important role in maintaining the structure and function of PELNs. Numerous studies have confirmed that PELNs contain lipids, with the main components being phosphatidic acid (PA), phosphatidylglycerol (PG), phosphatidylcholine (PC), phosphatidylinositol (PI), and phosphatidylethanolamine (PE) [27,47]. Some researchers have performed lipidomic analyses of PELN lipids from tomato fruit, grape, grapefruit, and tea. The results showed that purified PELNs are rich in lipids, and while the total lipid composition of PELNs from different sources may vary, these four lipids are still the main components. PELNs contain fewer types of proteins, which are mainly proteins regulating glycolipid metabolism and membrane-related proteins [28]. Previous studies have found large differences in the types and contents of proteins contained in various PELNs through proteomic assays. PELNs isolated from bitter orange, grapefruit, lemon, sweet orange, grapefruit, and *A. thaliana* were analyzed and found to contain a wide range of proteins. Additionally, the proteome of *A. thaliana* PELNs was enriched with proteins involved in coping with biotic and abiotic stresses [28,48]. PELNs contain a variety of nucleic acid components such as miRNAs, sRNAs, DNA, and other non-coding RNAs. Some researchers have found that PELNs from plants, such as blueberry, coconut, ginger, kiwi, orange, tomato, and Moringa oleifera, contain large amounts of 20–22 nt miRNAs [31,41,49]. More and more reports have shown that miRNAs not only perform biological functions within their original systems but also regulate transboundary gene expression [50]. In addition to the aforementioned biomolecules, active small-molecule components of homologous plants have also been detected in PELNs. PELNs, such as curcumin and capsaicin from ginger and green tea, can be used as non-toxic active ingredients that can inhibit or improve skin aging. Lycopene sulfones were detected in broccoli [51], and citric acid and citrulline were detected in lemon-derived ELNs [52]. Grapefruit-derived ELNs can detect naringin and naringenin [24].

## 4. Difference Between PELNs and Animal ELNs

In recent years, with the development of science and technology and people’s attention to health, the application of exosomes to the treatment of diseases has become a research hot spot. The study of animal-cell exosomes as carriers to transport drugs to target sites is more mature. Reports on PELNs from edible plants (e.g., medicinal plants) have gradually become the focus of recent research [53]. Due to their edible properties, extracted PELNs can be administered orally for cross-species targeting and gene expression regulation [54]. ELNs have also emerged as new, safe transport vectors, providing new research directions for drug delivery [55]. There is growing evidence that PELNs are structurally and functionally similar to animal-derived ELNs. Both types of nanoparticles promote the intercellular transport of active substances and facilitate signal transduction communication [56]. Additionally, they participate in RNA processing in both the nucleus and the cytoplasm. For example, ELNs extracted from plants such as ginger, ginseng, wheat, *Rhodiola rosea*, and licorice have antifibrotic, antiviral, and antitumor effects that are similar to those of animal-derived ELNs [57,58]. But there are also differences between animal ELNs and PELNs. ELNs from animal-cell sources require the culture of a large number of cells, have a low yield, and suffer from the problem of immunogenicity of the isoforms [59]. Compared with animal-cell-derived ELNs, PELNs have higher yields and shorter extraction cycles, are widely available, and have lower immunogenicity. PELNs also address the shortcomings of animal-derived ELNs, such as partial immunogenicity and limited sources [60]. In addition, certain animal-derived ELNs are easily recognized and killed by lysosomes [58]. The synthesis of liposomes, as well as modified exosomes, can have adverse effects, such as cellular stress and inflammatory vesicle apoptosis [61]. In contrast, edible PELNs not only have superior biocompatibility but also have less cytotoxicity to healthy tissues [62]. They can target specific tissues to treat diseases while reducing off-target effects through a special endocytosis mechanism. Therefore, they are expected to be used as candidate endogenous carriers for drug delivery [63].

## 5. PELN Isolation and Purification Techniques

**Figure 2:**
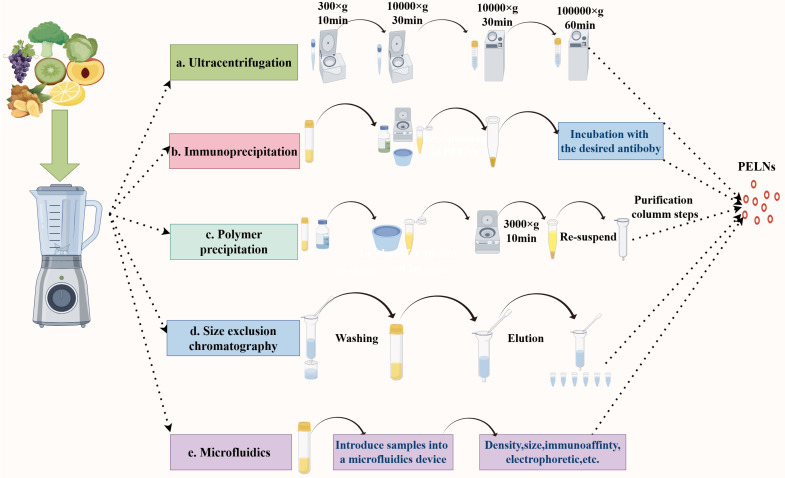
Isolation techniques for PELNs. (**a**) Ultracentrifugation; (**b**) Immunoprecipitation; (**c**) Polymer precipitation; (**d**) Size-exclusion chromatography; (**e**) Microfluidics.

Currently, techniques for extracting ELNs from animal-cell sources are relatively well established. However, extraction methods to obtain high-purity PELNs are relatively limited [64]. Before extracting PELNs, the active parts of the plant must be washed and then subjected to some simple treatments [65]. PELNs can be extracted from the seeds, roots, stems, fruits, leaves, and other parts of different plants [66,67,68]. Juicy fruits, such as grapefruit and strawberries, can be squeezed directly after washing to obtain an initial sample of PELNs [69]. Plant leaves and seeds, such as tea and coffee beans, require a vacuum infiltration centrifugation procedure in an infiltration buffer to obtain in vitro fluids before subsequent operations. The roots and stems of plants such as ginseng and ginger are hard and juicy, and a combination of a grinder and a juicer is required to extract PELNs from such plants, with the addition of an appropriate amount of PBS (phosphate-buffered saline) to the process [70]. The liquid obtained by filtration is subjected to subsequent extraction operations. Figure 2 shows the separation technique for PELNs. These methods include ultracentrifugation (Figure 2a) [66,67,68], immunoaffinity capture (Figure 2b) [71], polymer precipitation (Figure 2c) [72], size-exclusion chromatography (Figure 2d) [73], and microfluidic techniques (Figure 2e) [74]. Advances in these techniques have led to new insights into the structure and function of ELNs [75]. Currently, ultracentrifugation and sucrose density gradient centrifugation are the most commonly used ultracentrifugation techniques [76,77]. Combining ultracentrifugation with density gradient centrifugation and immunoaffinity capture techniques is commonly used for the isolation and extraction of PELNs [78]. However, each isolation and purification method has its advantages and disadvantages [65]. Table 2 lists several methods for extracting PELNs and their advantages and disadvantages.

## 6. Preservation of PELNs

Choosing the right extraction method based on different plant types is the first step toward the successful extraction of ELNs. Choosing the substrate used for ELN extraction and determining how to store them are crucial factors in determining the stability of ELNs [84]. ELNs have a bilayer lipid membrane vesicle structure, and their internal biomolecules must be protected from various enzymes in body fluids to maintain their integrity and bioactivity [85]. It is necessary to investigate preservation techniques for PELNs to protect the biological activity of ELNs and to make the transport and clinical use of ELNs easier. When extracting ELNs using various techniques, the suspension time of ELNs in PBS [86], the temperature, and the storage medium are all factors that affect the bioactivity and integrity of the obtained ELNs. Currently, in the field of ELNs of mammalian origin, there are various methods such as cryopreservation, lyophilization, and spray-drying preservation. However, no systematic research has been carried out on the preservation methods of plant ELNs, and the preservation techniques are limited [20].

Currently, the main PELN preservation techniques are freezing and drying [87]. Drying maintains the near-spherical shape of PELNs [88]. Cryopreservation keeps the temperature below the biochemical reaction temperature and maintains the functionality of the cells [89]. The commonly used storage temperatures for cryopreservation are 4 °C, −20 °C, and −80 °C. The isolated and purified secretion products can be stored at 4 °C for a short time. Storage at −20 °C and −80 °C for a long time is currently the most suitable preservation environment for various exosome samples. However, it is often difficult to maintain this condition during handling or transportation due to the low-temperature state [90,91,92,93,94,95,96]. However, the damage caused by the imbalance of medium osmolarity and the formation of intracellular ice crystals during the freezing process not only affects the labeling and biological characteristics of ELNs but may also lead to the accumulation of multilamellar vesicles and changes in their morphology and biological properties [97]. To prevent ELNs from repeated freeze–thawing, substances with strong water solubility and low toxicity, known as “cryo-protective agents,” are often added during the freezing–storage process [98]. Osmotic cryoprotectants with glycerol and ethylene glycol as the main components can effectively prevent the formation of ice crystals in cells. Non-penetrating cryoprotectants such as sucrose, trehalose, and lyophilization with trehalose are effective storing for ELNs for various applications [99,100]. There is no uniform understanding of the storage conditions and methods for ELNs. Therefore, during long-term storage, we need to find more suitable storage conditions for ELNs by comparing different storage methods.

## 7. Characterization of PELNs

At present, the identification of ELNs mainly includes ultrastructural structure detection, particle size detection, marker protein detection, and flow cytometry analysis [101]. The ultrastructural structure is typically inspected using transmission electron microscopy (TEM), scanning electron microscopy (SEM), or atomic force microscopy (AFM). These methods are commonly used to view the shape of ELNs, making them ideal for the identification of ELNs [102]. The morphological structure of PELNs observed under ultramicroscopic structure analysis was spherical or saucer-shaped [103,104]. Dynamic light scattering (DLS) and nanoparticle tracking analysis (NTA) are the most common methods for measuring ELN particle size and surface charge. NTA mainly detects the size and distribution of ELNs. This technology is easy to use, fast, robust, and accurate [105,106].

Flow cytometry analysis (FACS) is a method that has been developed in recent years for identifying PELNs [107]. This method mainly detects the number of ELNs. Western blot analysis mainly detects landmark proteins on the membrane surface of ELNs. The detection indicators of animal ELNs are usually CD63, CD81, and HSP90, but research on PELNs is immature in this area [108]. NTA and TEM, combined with transmission electron microscopy, are the classic identification methods for ELNs, regardless of their animal or plant origin. These methods can accurately and efficiently analyze the particle size distribution of ELNs, determining their size and quantity. The morphology and function of PELNs in different plants are listed in Table 3.

## 8. Functional and Biomedical Applications of PELNs

### 8.1. Functions of PELNs

#### 8.1.1. Participation in Substance Storage and Transportation

Studies have shown that wood such as spruce and poplar have exocrine functions that play important physiological roles in the xylem and phloem. The main function of these is to construct the xylem structure through the transport of glucanase in the xylem [118] and to participate in the storage and transportation of plant material.

#### 8.1.2. Transfer of Information Function

ELNs are membrane particles released by cells into their environment and are considered key players in intercellular communication [119]. Existing studies have shown that *A. thaliana* cells can deliver sRNAs into the host through the secretion of extracellular vesicles similar to ELNs. ELNs suppress the host’s immune response. These sRNA-containing vesicles aggregate at infected locations and are taken up by the fungal cells [120].

#### 8.1.3. Tuning Control of Intestinal Flora

The gut is the habitat of microbes, which are essential for many aspects of human biology, such as the immune system and metabolism. Through dietary intervention, alterations can be made to both the composition and activities of the microorganisms present in the gut [121]. Cells from an edible plant, which are both plant and mouse intestinal host cells, engage in interspecies communication [122]. In the lipid metabolism pathway, PELNs are more likely to be taken up by intestinal microbes [123]. These microbes regulate the structure and distribution of intestinal flora and the physiological activities of the body, especially in promoting the intestinal mucosal barrier function. This, in turn, alleviates colitis [124,125]. In recent years, many scholars have isolated and purified ELNs from Tartary buckwheat and have performed structural analyses and miRNA sequencing on the nanovesicles derived from Tartary buckwheat. ELNs (TB-ELNs) secreted by Tartary buckwheat cells contain RNA genetic material from the same plant. TB-ELNs also carry lipids, proteins, and other active components from Tartary buckwheat that can be delivered to the intestinal tract to exert their biological activities. Some miRNAs have important regulatory functions in *Escherichia coli* and *Lactobacillus rhamnosus*. They have a significant growth-promoting effect on these bacteria and increase the intestinal flora [126]. Bitter-almond-derived polyphenolic-extract-laden nanoparticles have bactericidal effects, and silver nanoparticles (AgNPs) were synthesized using a bitter-almond leaf extract as a reducing and capping agent [127]. Phytochemical screening experiments and FT-IR analysis of the leaf extracts of Staphylococcus bitter almond revealed the presence of phenolic, tannin, saponin, and flavonoid groups during the synthesis process. The antibacterial activities of the synthetic AgNPs against Gram-positive bacteria (*Streptococcus pyogenes* and *Staphylococcus aureus*) and Gram-negative bacteria (*Escherichia coli* and *Pseudomonas aeruginosa*) were evaluated, and a higher inhibition was observed in the district.

#### 8.1.4. Antiviral Effect

The involvement of PELNs in antiviral effects is based on the analysis of the composition of PELNs. It has been shown that *A. thaliana* PELNs are selectively enriched for specific miRNAs and small interfering RNA (siRNAs). Among the sRNAs identified, a class of “tiny RNAs” (10–17 nt) of unknown function was found to be highly enriched in PELNs [128]. PELNs were also successfully isolated from the extracellular fluid of sunflower seedlings. Compositional analysis showed that they contained cell-wall remodeling enzymes and defense proteins [129]. This resulted in the inhibition of fungal spore growth and cell death when the purified PELNs were used to treat a phytopathogenic fungus (*Sclerotinia sclerotiorum*) [130]. In addition, when barley (*Hordeum vulgare*) was infected with barley powdery mildew (*Blumeria graminis*), PELNs were enriched around the fungal aspirators. These structures not only prevented fungal infections but also inhibited hypersensitive cell death of the neighboring cells by closing the intercellular junctions [131,132]. When *A. thaliana* is infected with *Golovinomyces orontii*, the plant undergoes cell-wall thickening at the site of the pathogen sucker formation to inhibit the growth of the sucker and prevent it from robbing the plant cells of nutrients [133]. These findings suggest that PELN-mediated cross-border communication is an important mechanism for plant disease resistance.

### 8.2. Biomedical Applications of PELNs

#### 8.2.1. Anticancer Effects

**Figure 3:**
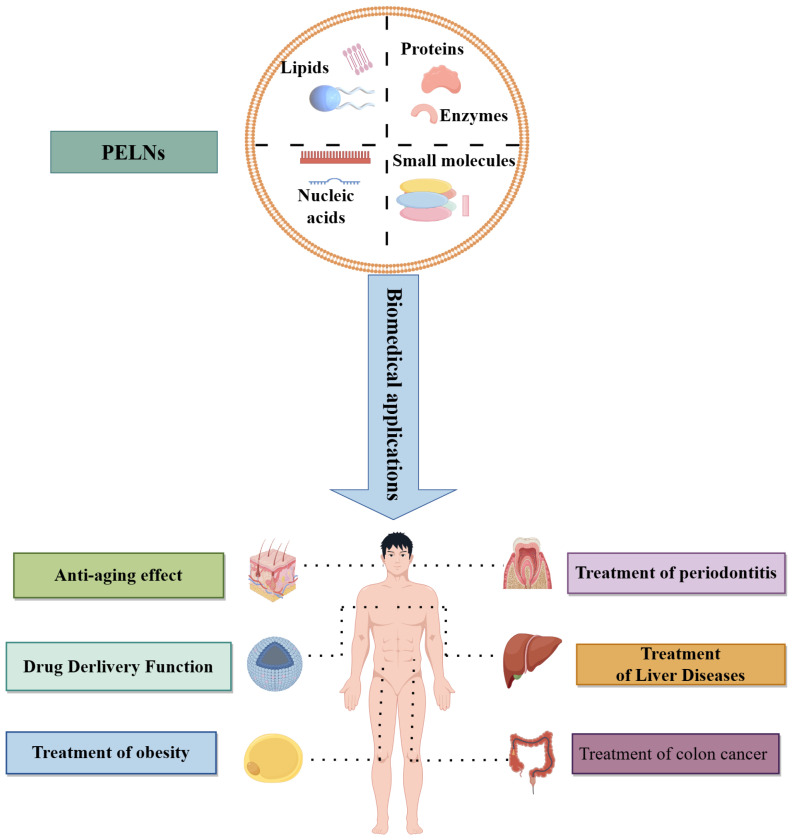
Biological applications of PELNs. Some of PELNs’ biological applications include anti-aging treatments [137], the treatment of periodontitis [138], drug delivery functions [137], the treatment of liver disease [139], the treatment of obesity [140], and the treatment of colon cancer [141].

Numerous studies have shown that PELNs act as communication mediators between cells and that PELNs can be involved in the therapeutic process of many diseases (Figure 3). Breast cancer is a common malignancy among women, and it has a high mortality rate compared with other tumors worldwide. PELNs have been isolated from fresh tea leaves. In vitro experiments showed that TLNTs could be efficiently phagocytized by breast cancer 4T1 cells. After two hours, the intracellular oxygen species (ROS) content was 5.8 times that of the 4T1 cells. High concentrations of reactive ROS can cause mitochondrial damage and cell cycle inhibition and eventually lead to tumor cell apoptosis [114]. Zhang et al. [113] isolated and purified vesicles (ACNVs) from the medicinal plant Asparagus (*Asparagus officinalis* L.) using a differential centrifugation method combined with sucrose gradient ultracentrifugation. The research was conducted in vivo and in vitro and revealed that ACNVs displayed the capability to notably restrain the growth of tumor cells without any negative consequences, and this was connected to the activation of the apoptosis pathway [134]. Exosome-like nanoparticles derived from lemons (LELNs) have been observed to impede the apoptotic cell death caused by cancer cell proliferation, as well as to impede the growth of colorectal cancer cells [135]. For the first time, vesicles were isolated from *Citrus limon* L. juice by ultracentrifugation [83]. In vitro experiments revealed that the nanovesicles isolated were capable of preventing cell death in numerous tumor cell lines by inducing TRAIL-mediated apoptosis and curbing cancer cell growth. We demonstrated that *Citrus limon* L. nanovesicles inhibited CML tumor growth in vivo by specifically reaching the tumor locations and activating TRAIL-mediated apoptotic cell processes. Grapefruit-derived microvesicles and nanovesicles showed different metabolomic profiles and anticancer activity in the A375 human melanoma cell line [136]. Ginseng-derived ELNs altered macrophage polarization to inhibit melanoma growth [30].

#### 8.2.2. Anti-Aging Effects

Melanocytes are neural-crest-derived cells that are localized in several organs in humans, including the epidermis, eye, inner ear, and soft meninges [142]. In recent years, plant-derived products have emerged as potential alternatives to chemical products due to their high antioxidant content and low side effects [143]. Lee et al. investigated the antimelanin effect of ELNs extracted from the leaves and stems of *Dendropanax morbifera* and found that leaf-derived ELNs and stem-derived ELNs reduced the melanin content and tyrosinase (TYR) activity in a concentration-dependent manner in a B16BL6 mouse melanoma cell line [144]. ELNs from certain plants may be novel natural substance candidates for use as antimelanin agents in cosmeceutical preparations.

#### 8.2.3. Treatment of Inflammation

Numerous studies have confirmed that natural ELNs from animals and PELNs can exert anti-inflammatory effects through interspecific communication and have important applications in the treatment of inflammatory bowel diseases [145].

##### Treatment of Colitis

Deng et al. conducted a study that showed that BDNs isolated from broccoli extract induced tolerogenic dendritic cells and protected mice from colitis through the adenosine-monophosphate-activated protein-kinase-mediated pathway. Oral administration of nanoparticles isolated from broccoli extract BDNs could protect mice from colitis [51]. BDNs regulate intestinal immune homeostasis through their targeting of dendritic cells (DCs), and BDN-mediated adenosine-monophosphate-activated protein kinase (AMPK) in DCs not only plays a role in the prevention of DC activation but also in inducing tolerant DCs. Oral administration of exosome-like nanoparticles extracted from tea tree tea can reduce the generation of ROS, decrease oxidative stress, inhibit the expression of inflammatory factors, and increase the expression of interleukin 10 (IL-10). This can repair the damaged colonic barrier, improve the structure of the intestinal flora, prevent and treat inflammatory bowel disease, and inhibit the occurrence and development of inflammatory colon cancer [27]. One study reported that a dietary plant-derived glucosylceramide (GlcCer) affected the levels of colonic factors in 1,2-dimethylhydrazine (DMH)-treated mice. The study also found that dietary GlcCer inhibited the formation of abnormal crypt lesions (ACF) and cytokine production in mice [146]. Wang et al. [13] isolated grapefruit juice nanovesicles from the pulp by sucrose gradient centrifugation and demonstrated that grapefruit-derived nanovesicles (GDNs) were selectively absorbed by intestinal macrophages, improving DSS-induced colitis in mice. GELN targeted intestinal stem cells, resulting in the remodeling of intestinal tissue and protection from DSS-induced colitis, as shown by Ju et al. [33]. Oral oat supplementation suppresses brain inflammation and improves memory function in alcohol-fed mice [147]. Apple-derived ELNs (ADNVs) were isolated and examined to determine their tumorigenic and anti-inflammatory effects on human cells such as macrophages and NCTC L929 cells. Microscopy and molecular biology techniques were used to characterize the ELNs and to measure cell proliferation, death, and miRNA levels, as well as to analyze gene expression and cellular uptake.

##### Prevention and Treatment of Gingivitis

Periodontitis is an infectious oral disease that causes the destruction of periodontal tissue and tooth loss [148]. Although great progress has been made in the treatment of periodontitis, effectively treating periodontal tissues invaded by periodontitis is still an urgent problem to be solved [138]. Therefore, finding new treatment methods for periodontitis is an urgent issue to be addressed. Natural products have good antibacterial, anti-inflammatory, antioxidative, and osteoprotective properties on periodontal tissues [125]. Some scholars have proven that a specific ratio of PA in ginger-derived nanoparticles (GELNs) can bind to the HBP35 protein on the surface of *Porphyromonas gingivitis*, thereby inhibiting the growth of bacteria and significantly reducing the incidence of gingivitis [31]. Ginger-derived ELNs are expected to be developed as a drug for the prevention and treatment of chronic periodontitis [57].

#### 8.2.4. Treatment of Obesity

Obesity and overweight are common modern health challenges [149]. It has been shown that adipose tissue secretes extracellular vesicles that communicate with peripheral cells and distant organs to regulate systemic metabolism [150]. Garlic ELNs reverse high-fat-diet-induced obesity via the gut/brain axis [34]. Sundaram et al. [31]. demonstrated that the oral administration of GaELNs resolved HFD-induced brain inflammation and obesity in a mouse model of HFD-induced obesity. GaELNs were preferentially taken up by microglial cells and inhibited brain inflammation through the IDO1-mediated AHR pathway and the c-Myc-mediated c-GAS/STING inflammatory pathway.

#### 8.2.5. Treatment of Liver Diseases

Several studies have reported on the role of exosomes in hepatitis B [151]. Ginger-derived nanoparticles protect against alcohol-induced liver damage [45]. Cannabis bud-derived ELNs, as hepatoprotective agents, attenuate liver fibrosis [152]. In addition, ELNs derived from *Pueraria lobata* can effectively alleviate alcohol intoxication, improve alcohol metabolism, inhibit iron death, and protect the liver from alcohol damage [153]. ELNs derived from garlic exhibit excellent anti-inflammatory effects, inhibiting NLRP3 inflammatory vesicle activation and inducing autophagy during liver injury [154]. Beta vulgaris-derived ELNs alleviate chronic doxorubicin-induced cardiotoxicity by inhibiting ferroptosis [155].

#### 8.2.6. Drug Delivery Function

Compared with traditional nanocarrier systems, PELNs have better biocompatibility, higher availability, greater safety, and lower toxicity, and can successfully deliver model drugs and target genes in various diseases [156]. PELNs naturally have the functions of cargo loading and long-distance delivery and, therefore, can be used to carry therapeutic agents and act as nanocarriers [157]. PELNs contain poorly soluble fractions that can improve their dispersibility and solubility, thereby enhancing their therapeutic effect.

Taxane compounds, such as paclitaxel and curcumin, are encapsulated in liposomes or polymer nanocarriers as enhancers. Alcohol, curcumin, and doxorubicin are commonly used methods [158]. However, synthetic liposomes or polymer nanocarriers not only have a low ability to penetrate biological barriers and to target delivery, but they also have low biocompatibility, poor stability, and high toxicity. In contrast, edible PELNs are safe, non-toxic, highly utilizable, and have good biocompatibility. They also have flexible dosing methods, including oral, transdermal, intratumoral, and nasal administration, for achieving drug delivery [159,160,161]. Ginger-derived lipids were used to create nanocarriers (GDNVs), and colon cancer cells were able to effectively take up GDNVs, demonstrating that GDNVs can efficiently load doxorubicin (Dox) and siRNA-CD98. Compared with the free drug, the targeted modified folate-coupled GDNV ligand mediated targeted delivery of Dox to Colon-26 tumors in vivo and enhanced the chemo-inhibition of tumor growth [162,163], leading to improved results. Simultaneously, orally delivered GDLVs were loaded with a very low dose of siRNA-CD98, which specifically and effectively reduced colonic CD98 gene expression. CELNs, which are exosome-like nanovesicles produced by celery, show excellent cell uptake efficiency. DOX was encapsulated into CELNs to construct engineered CELNs (CELNs-DOX). These were more effective than traditional synthetic vectors such as liposomes in treating tumors in vivo [156]. miRNAs were fully encapsulated in acerola cherry (*Malpighia emarginata* DC.)-derived exosome-like particles (AELNs), and the administration of an AELN/miRNA mixture in cells achieved the downregulation of miRNA target genes [164]. This method of directly packaging drugs in PELNs for oral delivery is expected to become a new strategy for treating various complex diseases. Drug delivery systems using nanotechnology show great promise in the treatment of diseases. Nanocarriers made from edible plants may be one of the safest drug delivery platforms in the future and have very high research value.

#### 8.2.7. Treatment of Neurodegenerative Diseases

Neurodegenerative diseases are a major cause of morbidity and disability and are receiving increasing attention due to their enormous impact on society [165,166]. The most common neurodegenerative diseases include AD and Parkinson’s disease (PD) [167]. Although several drugs are currently approved for the treatment of neurodegenerative diseases, the vast majority of them only help to alleviate the associated symptoms [167]. PELNs have shown therapeutic potential for neurological disorders. Studies have shown that oat ELNs can cross the blood–brain barrier (BBB), where their β-glucan cargo promotes uptake by microglia. Xu et al. demonstrated that ELNs derived from a medicinal plant, *Pueraria lobata* (Pu-ELNs), have an excellent ability to overcome cell membrane and endosomal barriers, ensuring the efficient delivery of adulterated biomacromolecule cargo to SH-SY5Y cells. On this basis, Pu-ELNs remove dysfunctional mitochondria via PINK1–Parkin-mediated mitochondrial autophagy and restore ATP replenishment by preserving the activity of mitochondrial respiratory chain complexes I and V, resulting in an overall improvement in mitochondrial dysfunction in SH-SY5Y cells [168]. It was also found that carrot-derived ELNs significantly reduced reactive ROS production and 6-hydroxydopamine-induced apoptosis in SH-SY5Y cells, suggesting that they are a potential new therapy for PD [78]. *Lycium ruthenicum Murray* (LRM)-derived ELNs (LRM-ELNs) inhibit AKT via the MAPK and PI3K/AKT signaling pathways to prevent Aβ-induced apoptosis in PC12 cells. LRM-ELNs have a protective effect in PC12 cells and can be considered a dietary supplement for alleviating neurodegenerative diseases. Potato-derived extracellular lipid nanoparticles (A-ELNs) were found to alleviate microglial inflammation. These A-ELNs have shown promise as therapeutic agents or drug delivery carriers for neuroinflammation [169]. These findings suggest that PELNs may have great potential in the treatment of neurological diseases.

#### 8.2.8. Antifibrosis Effects

Researchers studying animal ELNs have found that animal ELNs act as antifibrotic and anti-inflammatory agents in liver-like organs [170]. ELNs derived from various types of MSCs have shown therapeutic effects against fibrotic diseases in a variety of organs, including the heart, kidneys, and lungs. Similarly, when exploring the biological roles of PELNs, it was found that PELNs also act as antifibrotic agents. For example, ELNs derived from cannabis buds act as hepatoprotectors in attenuating liver fibrosis [152]. In addition, a small RNA (HJT-sRNA-m7) contained in the Chinese herbal medicine Rhodiola rosea can target proteins related to lung fibrosis, reduce the expression of fibrogenic factors, and improve the symptoms of pulmonary fibrosis [171]. Novel strategies of nanomedicine based on PELNs could provide potential benefits for the prevention and treatment of liver fibrosis [172].

#### 8.2.9. Application of PELNs in Cosmetics

The use of nanotechnology has been increasing day by day in recent years, and the cosmetic industry is one such area where it is being used extensively [173]. Peptides are a promising ingredient for skin care, but due to their inherent instability and the barrier function of the skin surface, their absorption and penetration into the skin is usually limited, which can severely hamper their skincare efficacy [174]. By encapsulating peptide-based cosmetic ingredients into engineered PELNs, the nanoscale PELN carrier significantly improves peptide penetration into the skin compared with free peptides [1]. Various nanocarriers are currently used in cosmeceuticals, with applications in skin care, hair care, oral care, etc. [175]. Nanocarriers have not only enhanced the efficacy of cosmeceutical products and provided better and longer-lasting results, but they have also expanded and grown the cosmetic industry and introduced nanotechnology into the cosmeceutical industry, creating an urgent need for scientific studies to investigate their efficacy, safety, and use [176,177].

#### 8.2.10. Antitumor Effects

Some studies have reported that certain PELNs can effectively inhibit tumor growth [178]. For example, PELNs derived from ginseng are rapidly recognized and internalized upon contact with macrophages. This induces M1-type polarization in macrophages, promoting the production of reactive ROS and triggering apoptosis in mouse melanoma cells. As a result, the growth of mouse tumors is inhibited. Raimondo et al. discovered that ELNs derived from lemons can be specifically targeted to the tumor site, effectively inhibiting the growth of multiple myeloma by activating TRAIL-mediated apoptosis in the tumor cells [29]. Multiple myeloma growth was inhibited by activating the apoptotic process mediated by the TRAIL signaling pathway [83]. The ability of grapefruit-derived naringenin from ELNs to inhibit the growth of human leukemia cells and bone marrow progenitor cells from leukemia patients has also been demonstrated [174].

## 9. Conclusions and Prospects

Currently, by isolating and identifying PELNs from various grains, fruits, and vegetables, the primary constituents of PELNs have been identified as proteins, nucleic acids, and lipids and their related small-molecule compounds. PELNs can be isolated through a range of techniques, including density centrifugation, polymer-based precipitation, ultrafiltration centrifugation, immunoaffinity capture, size-exclusion chromatography, microfluidic technology, and other methods. Cryopreservation and desiccation are two methods of storage. However, the conditions for storing PELNs are harsh. Factors such as temperature, time, and storage media can affect the biological activity and integrity of PELNs. In recent years, exosome research has developed rapidly from basic biology research to clinical applications. Although the current yield of PELNs is affected by the extraction technique, it is not possible to achieve the extraction of large quantities of high-purity PELNs. PELNs have a very high application value. PELNs have been used in the treatment of inflammation, lung cancer, tumors, and other diseases. PELNs have shown potential in treating human diseases. Even though PELNs from various plant sources have been employed as biotherapeutic agents or drug carriers for treating various human diseases, research on PELNs is still in its early stages. The extraction and identification methods of PELNs are relatively limited. The current isolation techniques for PELNs primarily rely on animal sources, resulting in high costs and low efficiency. There are few extraction and identification methods for PELNs. In addition, PELN membrane surface markers that have not yet been identified can only be distinguished through ultramicroscopic structure and particle size detection; thus, improving the efficiency of ELN detection is needed. Currently, the development of PELN technology with high separation purity, low cost, and easy operation, and the improvement of identification technology are urgent problems to be solved. PELNs originate from a multitude of sources and take many forms. The composition and size of PELNs from various plant sources vary, so standardizing the source of PELNs is advantageous for further research and utilization.

Much attention has been given to PELNs due to their impressive biological activity and antioxidative properties. The number of nanovesicles extracted from organic agricultural products is greater than that extracted from traditional agricultural products, and they have greater overall antioxidative effects. Oxidative ability and PELNs from organic agriculture, such as cereals, fruits, and vegetables, are expected to become a hot topic of PELNs research. As an emerging research area, PELNs face various problems and challenges as research continues in this area. PELNs exhibit many advantages in terms of biocompatibility, therapeutic capacity, targeting ability, and cellular uptake, and they can be developed into reliable drugs for the treatment of a wide range of common diseases through multidisciplinary cross-fertilization. With the aging of the population, the incidence of neurodegenerative diseases is increasing, which has a great influence on patients, families, and society. Exploring their pathology, early diagnosis, and early intervention has become the focus of problem-solving nowadays. The exploration of PELNs has found a great connection with neurodegenerative diseases. Although it is only in the research stage and its practical application still faces many difficulties, it is still a promising method.

In addition, when PELNs are used as therapeutic agents or drug carriers in the biomedical field, the safety, efficacy, stability, and controllability of the products are gold standards that must be met. However, the geographical distribution and seasonal variation of plants, as well as the stability and consistency of PELNs obtained from different regions and seasons, need to be systematically evaluated. With the maturing of soil-less culture technology, it is expected that plants will be brought from field cultivation to industrial production in the future. Plant tissue culture and cell culture technology will be used to provide continuous, stable, and consistent plant raw materials to control the quality of plant materials at the source and to improve the safety of plant materials. This may be the future trend in the application of PELNs.

## Figures and Tables

**Table 1:** Types of components of PELNs.

Plant Sources	Analytical Techniques	Component Types	Research Content or Function	Ref.
Lemon	RP-HPLC-ESI-Q-TOF-MS	Lipids (Phospholipids)	Anti-inflammatory effect through inhibition of the ERK1/2GNFGκB signaling pathway.	[29]
Ginseng	LC-MS/MS	Lipids (Ceramide)	Promotes the conversion of tumor-associated macrophages from M2 to M1.	[30]
Ginger	TLC; LC-MS/MS	Lipids (PA, PC)	Plays a role in maintaining the duration and amount of ELN accumulation in the gut; binds to heme-binding protein 35 (HBP35) on the surface of Porphyromonas gingivalis and reduces pathogenicity; promotes the migration of ELNs from the gut to the liver; lipids inhibit the activation of the NLRP3 inflammasome, which plays an important role in the regulation of inflammation.	[31,32]
Garlic, Grape	LC-MS/MS	Lipids (PA)	In BV2 cells, PA (36:4) binds to BASP1 to uptake garlic ELNs; PC inhibits activation of NLRP3 inflammatory vesicles. Plays an important role in inducing cell proliferation in colonic tissues.	[33,34]
Tea leaves	LC-MS/MS	Lipids (Galactose; (CH2O)6)	Promotes the accumulation of ELNs in colonic tissues of colitis mice.	[27]
Mulberry bark, Tea leaves, Garlic	LC-MS/MS	Proteins	Improves inflammatory bowel disease.	[27,35,36]
*Citrus clementina*	Shotgun proteomics	Proteins	Revealed the heterogeneous transport of ELN species.	[37]
Ginseng	SDS-PAGE	Proteins	Involved in the transformation of macrophage polarization.	
Broccoli	HPLC-MS/MS	Proteins	Water-channel proteins are associated with vesicle stability and permeability.	[38]
Bitter melon	Label-free	Proteins	Enhanced the therapeutic effect of 5G fluorouracil in oral squamous cell carcinoma.	[39]
Tomato	Nano high-performance liquid chromatography mass spectrometry	Proteins	Identification of the purity of tomato ELNs.	[40]
Soybean	Illumina HiSeq 2500 sequencing technology	RNAs (miRNAG5781)	Targets interleukin -17A to improve inflammation.	[41]
Ginger	----------	RNAs (Ath-miRNA-167a; aly-miRNA-159a; aly-miRNA-396a-5p, Rlcv-miRNA-rL1-28-3p)	Downregulates SpaC expression in Lactobacillus rhamnosus to ameliorate colitis; antagonizes cell attachment and invasion to prevent Porphyromonas gingivalis colonization by antagonizing epithelial cell attachment and invasion; inhibits SARS-CoVG2Nsp12 and spiking genes to ameliorate lung inflammation.	[32,42,43]
Apple	----------	Small molecules (Mdm-miRNA-7121d-h)	Interacts with solute carrier organic anion transporter family member 2B1 (SLCO2B1), downregulates gene expression of SLCO2B1, and affects human intestinal transporter protein function.	[44]
Ginger	----------	Small molecules (6-shogaol)	Role of 6-shogaol in activating nuclear factor red factor 2-related factor 2 (Nrf2) and ameliorating alcoholic liver injury in mice.	[45]
Broccoli	----------	Small molecules (Sulforaphane)	Sulforaphane activates AMP-activated protein kinase, leading to dendritic cell tolerance and ameliorating colitis in mice.	
Strawberry	----------	Small molecules (Anthocyanins, folic acid, flavonols, and vitamin C)	Prevention of oxygenation stress in BMSCs.	[46]

Abbreviations: MS, mass spectrometry; RP-HPLC-ESI-Q-TOF-MS, reversed-phase high-performance liquid chromatography coupled with electrospray ionization quadrupole time-of-flight mass spectrometry; SDS-PAGE, sodium dodecyl sulfate–polyacrylamide gel electrophoresis; LC-MS, liquid chromatography–mass spectrometry; BMSCs, bone marrow mesenchymal stromal cells.

**Table 2:** Comparison of extraction methods for PELNs from different plants.

Examples of Application	Extraction Method	Principle	Advantages	Limitations	Ref.
Carrot Ginseng	Ultracentrifugation	Particle density, size, or shape	Low cost, large sample size, and high throughput.	Low purity; repeated centrifugation causes damage to the cell structure.	[79,80]
Bitter melon Portulaca oleracea	Sucrose density gradient centrifugation	Exosome strip formation by centrifugation at ultra-high-speed using sucrose concentration gradients	Higher purity and ability to isolate exosome subpopulations.	High workload; time-consuming; low portability may disrupt the exosome structure; unable to distinguish between other impurities of the same density.	[15,81]
Lemon	Ultrafiltration centrifugation	Particle size and molecular weight	Broad separation range; a wide variety of commercial membranes.	Membrane contamination; product loss due to attachment to the membranes.	[82,83]
Broccoli	Size-exclusion chromatography	Particle size and molecular weight	Good separation effect; a wide variety of eluents.	Requires special equipment; long run time; difficulty in scaling production.	[26,78]
	Immunomagnetic bead method	Immune recognition and binding	The ability to isolate exosomes from specific sources allows for the isolation of subpopulations of exosomes of very high purity.	Inability to identify exosomes with different surface proteins; low yield, small sample size, and high cost; may damage the exosome structure and activity.	[26]
Grapes	Polymer precipitation	Hydrophilic polymers interact with the water molecules around exosomes, reducing the solubility of exosomes and forming precipitates.	Simple operation, no need for high-precision equipment; large sample volumes and high throughput.	Cumbersome sample preparation, low purity, and difficult-to-standardize procedures.	[13]
Grapefruit	Microfluidics	Isolation of exosomes according to various principles, such as immunological affinity, size, and density, is possible.	Automation, efficiency, and portability.	Complex equipment, lack of large-scale clinical sample testing, and small sample size are all challenges.	[72]

**Table 3:** Morphology and function of PELNs in different plants.

Plant Species	Morphological	Size (nm)	Functionality	Ref.
Barley	Ball-shaped	128.2	Promotes cell proliferation and wound healing	[109]
Buckwheat	Ball-shaped	141.8	Enhances intestinal microbial diversity, as well as increases levels of short-chain fatty acids	[110]
Cabbage mustard	Spherical or cup-shaped	32.4	Treatment of colitis	[51]
Cabbage	Ball-shaped	98.8–148.2	Enhances resistance of human cells to cell death and inflammation	[111]
Grapefruit	Ball-shaped	210.8 ± 48.62	Immunomodulation and anti-inflammation	[112]
Wine	Cup-shaped or spherical		Treatment of colitis	[33]
Asparagus	Cup-shaped	120	Inhibition of hepatocellular carcinoma cell proliferation	[113]
Tea	Teatox-like	166.9	Pro-apoptosis and microbiota regulation delay breast tumor growth	[114]
*Atractylodes lancea*	Orbicular	30–401	Inhibits melanin formation	[115]
Rose	Cup or sphere	75.51 ± 10.19	Increases expression of hematopoiesis-related transcription factors in vitro and in vivo	[116]
Coffee bean	Orbicular	40–100	Inhibition of the proliferative effect of cancer cells	[117]

## Data Availability

No new data were created or analyzed in this study. Data sharing is not applicable to this article.
